# Case report of a primary ovarian pregnancy in a primigravida

**DOI:** 10.4103/0974-1208.57231

**Published:** 2009

**Authors:** Subrat Panda, Laleng M Darlong, Santa Singh, Tulon Borah

**Affiliations:** Departments of Obstetrics and Gynecology, North East Indira Gandhi Regional Institute of Health and Medical Sciences, Shillong, Meghalaya, India; 1 Departments of Surgery, North East Indira Gandhi Regional Institute of Health and Medical Sciences, Shillong, Meghalaya, India

**Keywords:** Ovarian pregnancy, partial ovariectomy, primigravida

## Abstract

Primary ovarian pregnancy occurs quite rarely and that too usually in young highly fertile multiparous women using intra uterine device. We present a case where a young primigravida presented with abdominal pain and was diagnosed as ectopic pregnancy and was confirmed intra-operatively and histopathologically as primary ovarian pregnancy, managed with partial ovariectomy.

## INTRODUCTION

Primary ovarian pregnancy is a rare entity, the diagnosis of which continues to challenge the practicing clinicians. Since the first case reported by St. Maurice in 1689, many cases have been reported in the literature. Heartig estimated that ovarian pregnancy occurs in one in 25,000- 40,000 pregnancies.[1] Its frequency is 0.3-3.0 of all ectopic gestation.[2]

## CASE REPORT

**Figure 1:**
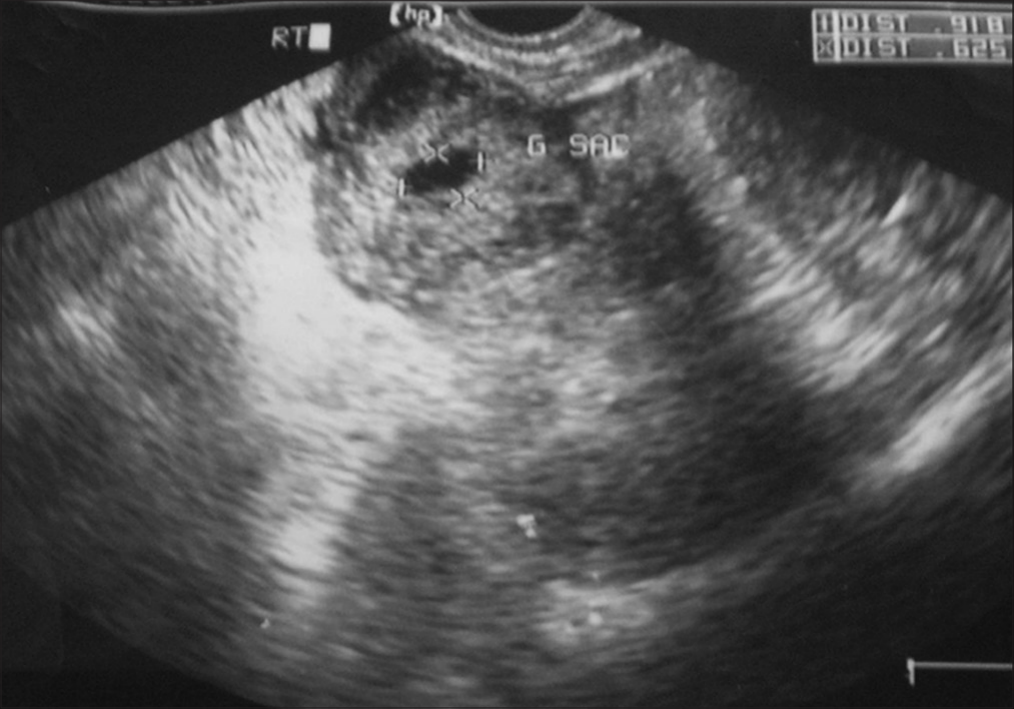
Ultrasonogram showing ectopic gestation

**Figure 2:**
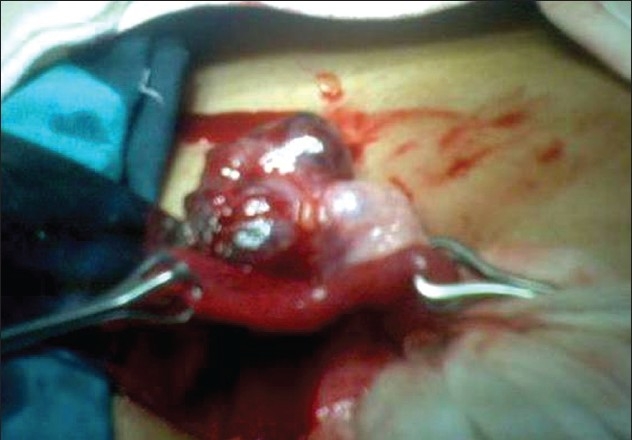
Intraop showing primary ovarian pregnancy

**Figure 3:**
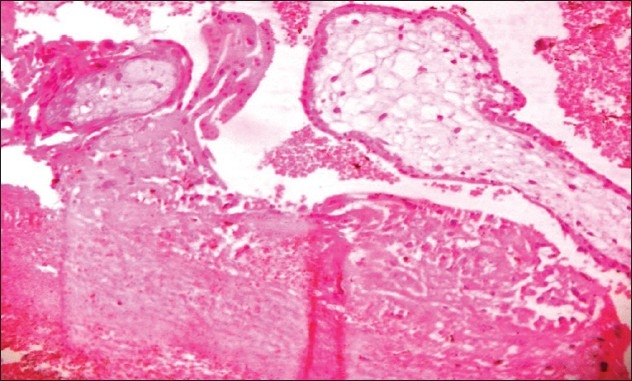
Histopathology slide showing trophoblastic villi and corpus luteum embedded in ovarian tissue

A 28-year-old primigravida with lower abdominal pain for 1 week with a history of 5 weeks amenorrhea was referred from the Department of Surgery. Her previous menstrual cycle was regular: average flow and no dysmenorrhea. On examination, no pallor, pulse 104/min, BP 100/70 mmHg and tenderness in the right iliac fossa were observed. Per vaginal examination showed normal uterine size and no cervical motion tenderness, but a palpable mass of size 4 cm × 4 cm was seen in the fornix. On investigation, the urine pregnancy test was positive, Hb% was 9.4 gm%, total leucocyte count was 10,800/cu mm, platelet count was 2.6 lakh and the blood group was A positive. On ultrasonography, no gestational sac was seen inside the uterus but a right adenexal gestational sac 1.1 cm × 0.5 cm × 0.5 cm was seen without a fetal pole or free fluid in the cul de sac [Figure 1]. Provisional diagnosis was unruptured ectopic pregnancy. Medical management with a single dose of methotrexate was offered to the woman. Serum β human chorionic gonadotropin (HCG) was 10,549 mIU/mL. On the fourth day of medical management, the patient complained of severe pain in the abdomen. The pulse rate was 120/min and the BP was 90/60 mmHg, following which a decision for laparotomy was taken because of the clinical features of shock (tachycardia, hypotension). However, in hemodynamically stable patients, such cases can be managed by laparoscopy if the facility and the expertise exist. Intra-operatively, the uterus was normal in size, with no hemoperitonium and both fallopian tubes were normal. The right ovary was enlarged with a bluish red mass of size 4 cm × 4 cm, with oozing of blood from the surface of the mass [Figure 2]. Right-sided partial ovariectomy was performed and the contents were evacuated and sent for histopathology. The postoperative period was uneventful. On histopathological examination, trophoblastic villi and corpus luteum embedded in the ovarian tissue were seen, which were confirmatory of primary ovarian pregnancy [Figure 3]. Thus, the intra-operative findings and the histopathology examination satisfied the criteria for ovarian pregnancy as described by Spigelberg,[3] which is as follows: (a) intact fallopian tube on the affected side, (b) fetal sac must occupy the position of the ovary on the affected side, (c) ovary connected to the uterus by ovarian ligament, (d) ovarian tissue must be located in the sac wall, which was confirmed by histopathology.

## DISCUSSION

Primary ovarian pregnancy is one of the rarest types of extra-uterine pregnancy. With few exceptions, the initial diagnosis is made on the operating table and the final diagnosis only on histopathology on the basis of the four Spigelberg criteria, establishing that the pregnancy is limited to the ovary and does not involve the tube.[3]

The cause of primary ovarian pregnancy remains obscure. Borrow concluded that chance is a reasonable explanation of ovarian pregnancies.[4] Other hypotheses have suggested interference in the release of the ovum from the ruptured follicle, malfunction of the tubes and inflammatory thickening of the tunica albugenia. Current intra uterine contraceptive device used may also be a cause.[5] The entity, empty follicle syndrome, where no oocytes are retrieved from the mature ovarian follicles with apparently normal follicular development and estradiol levels, after controlled ovarian hyperstimulation for an assisted reproductive technology cycle, despite repeated aspiration and flushing, can also be a cause for primary ovarian pregnancy.[6]

The signs and symptoms of ovarian pregnancy are similar to disturbed tubal pregnancy, conditions most commonly confused with ruptured hemorrhagic corpus luteum and chocolate cyst or tubal ectopic pregnancy.[5] Rupture in the first trimester is the usual rule in an ovarian ectopy, but the pregnancy may advance to full term.[7]

With the improvement in the ultrasonographic skill and instrumentation, especially with the use of vaginal probe, ovarian pregnancy can be diagnosed pre-operatively.[8]

No case of repeat ovarian pregnancy has been reported in contrast to approximately 15% recurrent tubal pregnancy.[9] Treatment consists of a single dose methotrexate protocol or conservative surgery. More preferred is partial overiectomy by either laparotomy or laparoscopy.[10] Methotrexate treatment in women with tubal ectopic pregnancy shows a success rate of > 82%, with the beta HCG level between 10,000 and 14,999 mIU/mL,[11] but according to the American Society of Reproductive Medicine guidelines, a beta HCG level more than 5000 mIU is a relative contraindication to medical therapy.[12] As in our case, the woman was a primigravida, with gestational sac size less than 2 cm, no cardiac activity, hemodynamic stability and no hemoperitonium. We had started medical therapy with methotrexate under strict supervision. Fertility after ovarian pregnancy remained unmodified.[13]

## CONCLUSION

The diagnosis of ovarian pregnancy is difficult. Thus, it continues to challenge the practicing clinicians. Now, with ultrasonographic advances, it can be diagnosed early, leading to conservative treatment and preservative surgery.
